# Sirtuins as Potential Therapeutic Targets for Mitigating Neuroinflammation Associated With Alzheimer’s Disease

**DOI:** 10.3389/fncel.2021.746631

**Published:** 2021-09-22

**Authors:** Kurukulasooriya Kavindya Madushani Fernando, Yasanandana Supunsiri Wijayasinghe

**Affiliations:** Department of Biochemistry and Clinical Chemistry, Faculty of Medicine, University of Kelaniya, Ragama, Sri Lanka

**Keywords:** Alzheimer’s disease-AD, neuroinflammantion, microglia, NF-kB, NLRP3 inflammasome, sirtuins (SIRT), sirtuin activators

## Abstract

Alzheimer’s disease (AD) is the most common neurodegenerative disorder, which is associated with memory deficit and global cognitive decline. Age is the greatest risk factor for AD and, in recent years, it is becoming increasingly appreciated that aging-related neuroinflammation plays a key role in the pathogenesis of AD. The presence of β-amyloid plaques and neurofibrillary tangles are the primary pathological hallmarks of AD; defects which can then activate a cascade of molecular inflammatory pathways in glial cells. Microglia, the resident macrophages in the central nervous system (CNS), are the major triggers of inflammation; a response which is typically intended to prevent further damage to the CNS. However, persistent microglial activation (i.e., neuroinflammation) is toxic to both neurons and glia, which then leads to neurodegeneration. Growing evidence supports a central role for sirtuins in the regulation of neuroinflammation. Sirtuins are NAD^+^-dependent protein deacetylases that modulate a number of cellular processes associated with inflammation. This review examines the latest findings regarding AD-associated neuroinflammation, mainly focusing on the connections among the microglial molecular pathways of inflammation. Furthermore, we highlight the biology of sirtuins, and their role in neuroinflammation. Suppression of microglial activity through modulation of the sirtuin activity has now become a key area of research, where progress in therapeutic interventions may slow the progression of Alzheimer’s disease.

## Introduction

The number of people living with different stages of dementia is growing rapidly, making it a major public health issue all over the world (Rizzi et al., 2014). In 2018, there were around 50 million people with dementia globally, and this number is projected to rise by threefold, reaching 152 million by 2050 (Patterson, 2018).

Alzheimer’s disease (AD) is the leading cause of dementia in the elderly, accounting for 60–80% of all dementia cases worldwide (Erkkinen et al., 2018; The Alzheimer’s Association, 2020). Several contributing factors have been identified, with older age considered the most important known risk factor for the development of AD (Hou et al., 2019; The Alzheimer’s Association, 2020). One in ten individuals above age 65 are reported to be suffering from AD and disease prevalence continues to rise with the increase in life expectancy (Hou et al., 2019). Interestingly, while the population over age 65 is projected to grow from 9 to 16%, the number of people over the age of 80 years is projected to triple by 2050 (United Nations, 2019). This aging-associated cognitive impairment is known to occur due to a range of structural and functional alterations in the brain of primates that occur progressively over time (Peters, 2002). These age-related brain changes include accumulation of misfolded proteins (Yankner et al., 2008; Jucker and Walker, 2013), impairment of adult hippocampal neurogenesis (Spalding et al., 2013; Ernst and Frisén, 2015; Moreno-Jiménez et al., 2019), and persistent low-grade inflammation (Yankner et al., 2008; Lull and Block, 2010; Jucker and Walker, 2013). In this review, we examine the recent advances in several aspects of molecular neuropathology related to neuroinflammation and its implication to AD with special emphasis on the role of sirtuins during microglial immune response and sirtuin activators as promising neuroprotective agents for AD.

## Molecular Pathology of Alzheimer’s Disease

AD is an irreversible, severe neurodegenerative disorder, which is clinically characterized by a gradual decline in memory and other cognitive functions (Scheltens et al., 2016). The pathological brain changes typically begin much earlier than the onset of AD clinical symptoms; changes that include the deposition of β-amyloid (Aβ) peptides outside the neurons (i.e., senile or Aβ plaques) and the intraneuronal accumulation of an abnormal form of tau protein (i.e., neurofibrillary tangles, NFTs) (Sperling et al., 2011; Kumar et al., 2015; Hanseeuw et al., 2019).

Aβ pathology arises from the proteolytic cleavage of a large transmembrane glycoprotein called amyloid precursor protein (APP) by β- and γ- secretases, resulting in 37–49 amino acid peptides known as Aβ monomers (Zheng and Koo, 2011; Chen et al., 2017). APP is abundantly expressed in the brain and performs a range of signaling functions in neuronal development, synaptic maintenance, and neuronal homeostasis (van der Kant and Goldstein, 2015). Aβ monomers are intrinsically disordered peptides and aggregate to form insoluble oligomers, fibrils, and plaques. The Aβ_1__–__4__2_ (Aβ_42_) fibril is the predominant protein form in the senile plaques of AD. Aβ_42_ fibril aggregates rapidly and is more neurotoxic than the other Aβ species (Colvin et al., 2016; Wälti et al., 2016). A progressive extracellular Aβ accumulation has been identified mainly in the cerebral cortex and hippocampus of APP transgenic mouse models (Sasaguri et al., 2017). On the other hand, the Tau pathology in AD is only observed several years after the initiation of Aβ aggregation (Musiek and Holtzman, 2015; Sasaguri et al., 2017). Tau is a microtubule-associated phosphoprotein that stabilizes neuronal microtubules and hence serves an essential role in axonal transport. An aberrant hyperphosphorylation of tau protein causes its disengagement from microtubules and subsequent formation of filamentous aggregates called neurofibrillary tangles (Iqbal et al., 2010; Wang and Mandelkow, 2016). The intracellular accumulation of NFTs leads to the loss of neuronal function and eventual to neuronal death.

In addition, dysregulation of brain cholesterol metabolism has also been found to be associated with the development of AD (Feringa and van der Kant, 2021). The accumulation of cholesterol in neurons promotes the interaction of APP with β- and γ-secretases in lipid clusters (lipid rafts) in neuronal membrane, resulting in Aβ formation (Di Paolo and Kim, 2011). It was recently shown that cholesterol and apolipoprotein E (apoE) derived from astrocytes play an important role in amyloid plaque deposition through the regulation of membrane lipid raft function (Wang et al., 2021). Moreover, genetic variation in apoE, a cholesterol transport protein is identified as a risk factor for late-onset AD (Corder et al., 1993).

## Neuroinflammation and Alzheimer’s Disease

In addition to misfolded protein aggregates, neuroinflammation plays a crucial role in the pathogenesis and progression of Alzheimer’s disease (Heneka et al., 2015; Heppner et al., 2015; Calsolaro and Edison, 2016; Hampel et al., 2020). Neuroinflammation is a complex innate immunological response driven by microglia and astrocytes in the central nervous system (CNS) (Colombo and Farina, 2016; Ransohoff, 2016), a response which appears to play a dual role in brain health. While CNS inflammation is neuroprotective in the acute phase response, it becomes detrimental when progressing into a chronic phase (Kinney et al., 2018). Aging is known to be associated with high levels of proinflammatory mediators and increased microglial activity, resulting in chronic inflammation in the brain (i.e., neuroinflammation) and increased blood-brain barrier (BBB) permeability (Schuitemaker et al., 2012; Elahy et al., 2015). Aged microglia show altered morphology and function, particularly impaired phagocytosis, proteostasis, motility, and migration (Mosher and Wyss-Coray, 2014). These effects are further aggravated by misfolded protein deposits in AD. Aβ and tau deposition in/around the vasculature in the AD brain triggers proinflammatory and cytotoxic events, resulting in increased BBB permeability that exacerbates the neurodegenerative process and neuroinflammation (Zenaro et al., 2017). Microglial activation is positively correlated with both amyloid deposition and tau aggregation in patients with mild cognitive impairment (MCI) and AD (Parbo et al., 2017; Dani et al., 2018; Nordengen et al., 2019). The activated microglia are identified primarily surrounding the Aβ plaques (Heneka et al., 2015; Navarro et al., 2018).

Aβ activates microglia and induces the production of a plethora of inflammatory mediators, such as proinflammatory cytokines (e.g., TNF-α, IL-1β, IL-6), chemokines, nitric oxide (NO), and prostaglandins (e.g., PGE_2_) that promote neuronal death (Glass et al., 2010). This inflammatory response has also been suggested as a contributing factor for the development of NFT in AD (Kitazawa et al., 2004; Kinney et al., 2018). In addition, the peripheral levels of several inflammatory cytokines, including interleukin (IL)-1, IL-6, and tumor necrosis factor (TNF)-α are often elevated in patients with dementia (Koyama et al., 2013; Lai et al., 2017; Darweesh et al., 2018; Shen et al., 2019). Hence, the use of non-steroidal anti-inflammatory drugs (NSAIDs) in the prevention of AD has been investigated, with the initial observations having variable outcomes (Miguel-Álvarez et al., 2015; Benito-León et al., 2019). However, since the neuroinflammation begins very early in AD pathology, the administration of NSAIDs at the preclinical stage of AD is proposed to be beneficial (Cuello, 2017).

## Role of Microglia in Alzheimer’s Disease-Associated Neuroinflammation

Microglia are innate immune cells residing in the CNS that are derived from myeloid cells in the yolk sac and enter the CNS during early embryonic development (Ginhoux et al., 2010; Kettenmann et al., 2011). Their main function is to efficiently safeguard the CNS from pathologic insults (Wolf et al., 2017). In the healthy brain, microglia appear as ramified cells with small somas, and they exist in a “resting” or homeostatic state while constantly surveying the brain environment. In addition, microglia perform a number of housekeeping functions, including neurogenesis, maintenance of neuronal plasticity, synaptic connectivity, regulation of cognitive functions, and stochastic scanning of normal CNS with their fine branches (Nimmerjahn et al., 2005; Ji et al., 2013; Parkhurst et al., 2013). Microglia become activated in response to danger signals such as pathogens and brain injury. Activated microglia acquire an amoeboid morphology and respond to stimuli by activating phagocytosis and inflammatory (anti-inflammatory or proinflammatory) signaling pathways; responses that depend on the nature of the inflammatory microenvironment (ElAli and Rivest, 2016; Minter et al., 2016; Salter and Stevens, 2017). Microglia are demonstrated to be increasingly activated both in humans and in mice during aging (Sierra et al., 2007; Cribbs et al., 2012) and all of the innate immune system pathways, including genes associated with toll-like receptor signaling and inflammasome activation have been found to be upregulated in the aging brain (Cribbs et al., 2012).

Microglia can be activated by a number of stimuli, such as bacterial cell wall lipopolysaccharides (LPS), pesticides (e.g., paraquat), misfolded proteins (e.g., Aβ, α-synuclein), and air pollutants (Lull and Block, 2010). A variety of cell surface receptors are involved in the microglial response, including toll-like receptors (TLRs), NOD-like receptors (NLRs), receptors for advanced glycation end products (RAGE), scavenger receptors, formyl peptide receptors, complement receptors, and Fc receptors (Doens and Fernández, 2014). In Alzheimer’s disease, microglia interact with different Aβ species via TLRs (e.g., TLR2, TLR4, TLR6, TLR9), scavenger receptors (e.g., SR-A1, CD36, α6β1 integrin, CD47), RAGE, and complement receptors (e.g., CR3) (Doens and Fernández, 2014; Fiebich et al., 2018). TLRs are pattern recognition receptors (PRRs) that serve as the front-line defense against tissue injury and infection (Kigerl et al., 2014). PRRs identify molecules associated with microorganisms (pathogen-associated molecular patterns, PAMPs). In addition, TREM2 (triggering receptor expressed on myeloid cells 2) and CD33 receptors have also been found to be associated with the microglial immune response and pathogenesis of AD (Yeh et al., 2017; Griciuc et al., 2019).

Microglial activation during CNS inflammation displays both beneficial and detrimental effects, which is known as a Janus face (Gomes-Leal, 2019). After an acute brain injury (e.g., ischemia, stroke), microglia elicit a neuroprotective immune response that removes the pathogens/cell debris and promotes tissue repair (Neumann et al., 2008; Badoer, 2010). Insufficient microglial clearance of tissue debris opens the pathway of development of several neurodegenerative diseases (Neumann et al., 2009). During normal aging and low-grade systemic inflammation (e.g., as seen in obesity, atherosclerosis), microglia undergo priming (or sensitization) that results in vigorous microglial response to secondary inflammatory stimuli (Perry and Teeling, 2013; Niraula et al., 2017).

Perry and colleagues showed that systemic inflammation characterized by elevated serum TNF-α and IL-6 is associated with increased cognitive decline and other neuropsychiatric symptoms found in AD, suggesting that the enhanced cytokine response in the brain is due to higher sensitivity of primed microglia to systemic inflammatory signals (Holmes et al., 2011). Moreover, the primed microglia found to produce exaggerated IL-1β response to LPS-induced secondary inflammation in APP/PS1 transgenic mouse model of AD. It was further highlighted that IL-1β primes the astrocytes to produce increased levels of IL-6 and chemokines such as CCL2, CXCL1, and CXCL10 (Lopez-Rodriguez et al., 2021). Similarly, elevated IL-1β and IL-6 levels have also been observed in the brain of AD patients who died with systemic infection (Lopez-Rodriguez et al., 2021). These results demonstrate that microglia-astrocyte crosstalk intensifies the inflammatory responses to acute stimulation leading to cognitive dysfunction. Readers are encouraged to read the review article by González-Reyes et al. (2017) for the role of astrocytes in neuroinflammation and AD.

Accumulating evidence demonstrates that a subset of activated microglia respond to Aβ by clustering at the site of lesion (Keren-Shaul et al., 2017; Shahidehpour et al., 2021). These microglia are known as disease-associated microglia (DAM). DAM perform protective role early in the disease by clearing Aβ that involves TREM2 signaling pathway. TREM2 is an immunoreceptor specific to microglia and is important for phagocytosis and for suppression of inflammatory response in microglia (Keren-Shaul et al., 2017; Ulland and Colonna, 2018). Rare genetic variants of TREM2 have been identified to impair microglial function and hence identified as risk factors for late-onset AD (Gratuze et al., 2018). It was also reported that replicative senescence of microglia leads to formation of DAM and contributes to early Aβ pathology (Hu et al., 2021).

**FIGURE 1:**
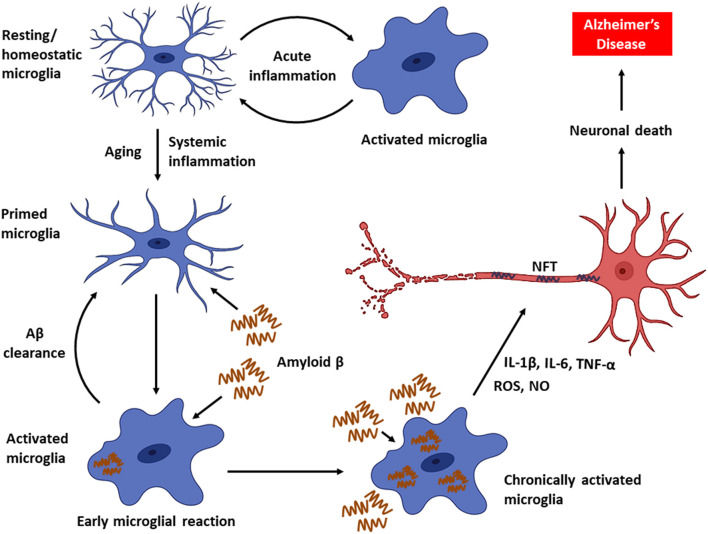
Microglial activity in health and disease. Microglia, the primary innate immune cells in the CNS elicit a protective immune response during an acute brain injury. Microglia are primed with aging. Chronic activation of microglia by Aβ triggers the persistent release of proinflammatory mediators, which can eventually cause neuronal death leading to AD.

As the disease progresses, a decline in microglial phagocytic efficiency has been observed due to decreased expression of the Aβ-binding SR-A1, CD36, and RAGE, as well as the enzymes that degrade Aβ (Hickman et al., 2008). Thus, accumulation of Aβ in DAM drives neuroinflammatory responses by producing a plethora of proinflammatory mediators (e.g., TNF-α, IL-1, and IL-6) and neurotoxic molecules (e.g., nitric oxide, superoxide), leading to accelerated AD progression (Block et al., 2007; Hickman et al., 2008; Smith et al., 2012; Deczkowska et al., 2018). Furthermore, intracellular accumulation of Aβ leads to microglial death, and the Aβ that is released from dying microglia further contribute to the formation and progression of Aβ plaques in the brain (Baik et al., 2016). In fact, the impairment of microglial function found to occur early in the course of AD in an amyloid-dependent manner in mouse models, and the phagocytic capacity of microglia, could be re-established by lowering the Aβ burden (Krabbe et al., 2013). In addition, persistent activation of microglia also promotes the hyperphosphorylation of tau and consequent development of NFT (Kitazawa et al., 2004). Chronic activation of microglia results in an exacerbation of AD pathology and progressive brain damage (Figure 1). Hence, the suppression of microglial activity has been considered as a potential treatment strategy against AD.

## Microglial Inflammatory Pathways

Gomes-Leal proposed “friendly fire hypothesis” to explain the Janus face of microglia. It is possible that microglia employ the same biochemical tool kit used to fight against pathogens even in the absence of infections, resulting in friendly fire death of neurons (Gomes-Leal, 2019). Aβ are endogenous molecules that act as danger associated molecular patterns (DAMPs) and can be sensed by microglial TLRs, which are usually used to detect, fight, and eliminate pathogens during infections. The binding of Aβ to TLRs activates microglia and triggers the production of proinflammatory cytokines. Aβ-bound TLR initiates a number of signaling cascades through myeloid differentiation factor 88 (MyD88) leading to activation of transcription factors, including nuclear factor kappa B (NF-κB) (Kawai and Akira, 2007). NF-κB is considered the central regulator of inflammation, which drives the expression of cytokines, chemokines, inflammasome components, and adhesion molecules (Liu et al., 2017). NF-κB exists in multiple forms, with the heterodimer of p65 (RelA) and p50 subunits (p65/p50) being the most prevalent species. In the inactive state, NF-κB is sequestrated in the cytoplasm through interactions with the inhibitor proteins of κB (IκB). Upon stimulation, IκB gets phosphorylated by IκB kinase and facilitates its ubiquitination and proteasomal degradation, enabling the active NF-κB entry into the nucleus to elicit transcriptional activity (Zhang Q. et al., 2017). In addition, NF-κB activity is also modulated by protein modifications, such as phosphorylation, acetylation, methylation, etc. (Huang et al., 2010).

**FIGURE 2:**
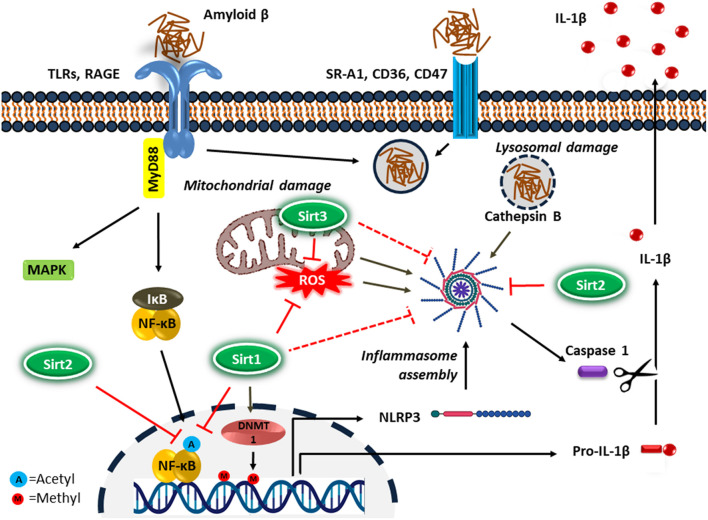
Microglial inflammatory response in Alzheimer’s disease. Microglia detect the presence of Aβ by a number of cell surface receptors, which trigger the microglial inflammatory response through activation of the proinflammatory transcription factor NF-κB and NLRP3 inflammasome. Sirtuins, particularly Sirt1-3 inhibit neuroinflammation in AD possibly by suppressing NF-κB and NLRP3 inflammasome activity.

NF-κB upregulates the transcription of pro-IL-1β and cytoplasmic pattern recognition receptor NLR (nucleotide-binding oligomerization domain leucine-rich repeat containing receptor or NOD-like receptor) family pyrin domain containing protein 3 (NLRP3) in macrophages (Bauernfeind et al., 2009). IL-1β is a key proinflammatory cytokine associated with the inflammatory response in AD (Shaftel et al., 2008). In addition, DNA methylation is a common epigenetic modification that inhibits gene transcription. It has been reported that the age-dependent elevation of IL-1β transcription in microglia is associated with selective hypomethylation of IL-1β promoter in humans (Cho et al., 2015). On the other hand, since the IL-1β is synthesized as its inactive precursor, pro-IL-1β, maturation of IL-1β requires the activity of caspase-1, which is an intracellular proinflammatory caspase (Halle et al., 2008). Caspase-1 also exists as an inactive pro-caspase-1, which is proteolytically activated by a multiportion assembly called inflammasome, in particular the NLRP3 inflammasome in microglia. The inflammasomes are responsible for sensing potential threats and inducing an inflammatory response. The NLRP3 inflammasome consists of the NLRP3 receptor, ASC (apoptosis-associated speck-like protein containing a CARD) adaptor, and caspase-1 protease and is the most studied inflammasome in neurodegenerative disorders. A large number of stimuli can activate NLRP3 inflammasome through promoting the assembly of inflammasome components (Figure 2). Diverse endogenous danger signals, such as K^+^ efflux, reactive oxygen species (ROS), mitochondrial and phago-lysosomal damage (He et al., 2016) and pathogen-derived signals, such as virus, bacteria, fungi, parasites (Zheng et al., 2020) interact with NLRP3 receptor component and trigger its oligomerization, resulting in ASC binding and activation of caspase-1.

Elevated expression of active caspase-1 has been found in patients with MCI and AD (Heneka et al., 2013). NLRP3 receptor expression is also under the control of transcription factor NF-κB (transcriptional priming of NLRP3) (Bauernfeind et al., 2009). It was recently reported that oligomeric and fibrillary Aβ could directly activate the NLRP3 inflammasome in microglia, resulting in activation of caspase-1 (Nakanishi et al., 2018; Lučiūnaitė et al., 2020). Furthermore, NLRP3 inflammasome has been found to be important for the development and spread of Aβ pathology in mice (Venegas et al., 2017).

Recently, NLRP3 inflammasome activation in microglia was also shown to drive tau pathology in mice (Ising et al., 2019). Furthermore, microglia contribute to tau pathology through IL-1 mediated pathological phosphorylation of tau protein via the p38 mitogen-activated protein kinase (p38-MAPK) pathway in primary microglial cells activated by Aβ or LPS in neocortical neuron cultures (Li et al., 2003). In addition, LPS induced inflammation has been demonstrated to trigger hyperphosphorylation of tau by a cyclin-dependent kinase 5 (cdk5) mediated pathway in a transgenic mouse model of AD (Kitazawa et al., 2005). IL-6 has also been proven to induce the abnormal phosphorylation of tau protein in hippocampal neurons by deregulating the cdk5 pathway (Quintanilla et al., 2004).

## Sirtuin Biology

Sirtuins (Sirts) are a family of evolutionary conserved enzymes with protein deacetylase/deacylase activity that depend on nicotinamide adenine dinucleotide (NAD^+^) (North and Verdin, 2004). They were first discovered as genetic silencing factors (i.e., silent information regulator, Sir) in yeast that extends the replicative life span by histone deacetylation, resulting in heterochromatin formation and subsequent transcriptional repression of mating-type genes (Kaeberlein et al., 1999; Imai et al., 2000). Subsequently, Sirts were identified as a critical factor of aging and longevity control in a number of other organisms, including worms, flies, and mice (Imai and Guarente, 2016). Moreover, Sirts are also linked to caloric restriction-mediated slowing of aging and lifespan extension in mammals (Bishop and Guarente, 2007; Guarente, 2013).

Seven sirtuins (Sirt1-7) have been identified in mammals so far. They possess various enzymatic activities and are present in different compartments within the cell (Table 1). All mammalian Sirts contain a conserved catalytic core region surrounded by variable N- and C-terminal extensions. The catalytic core consists of a large NAD^+^ binding Rossmann-fold domain and a smaller zinc-binding domain. The variable N- and C- terminal regions appear to be involved in substrate recognition and in regulation of sirtuin activity (Feldman et al., 2012). Sirts deacetylate/deacylate both histone and non-histone proteins (e.g., p53, Ku70, FOXO, PPARγ, PGC-1α, NF-κB, tubulin, MnSOD, etc.). In addition, some mammalian Sirts (e.g., Sirt4, Sirt6) possess NAD^+^-dependent ADP-ribosyltransferase activity, which transfers ADP-ribose from NAD^+^ to protein substrates (Michan and Sinclair, 2007). Since sirtuins modify multiple proteins, they can regulate cellular processes as diverse as energy metabolism and aging (Michan and Sinclair, 2007; Haigis and Sinclair, 2010; Houtkooper et al., 2012).

**TABLE 1:** Subcellular location and enzyme activity of sirtuins and their potential effects on Alzheimer’s disease.

Sirtuin	Subcellular location	Enzyme activity	Possible effects on AD	References
Sirt1	Nucleus, cytoplasm	Deacetylase	Non-amyloidogenic APP processing, prevent tau aggregation, reduce oxidative stress, promote mitochondrial biogenesis, anti-inflammatory	Brunet et al., 2004; Yeung et al., 2004; Yuan Y. et al., 2016; Min et al., 2018; Zhang et al., 2020
Sirt2	Cytoplasm, nucleus	Deacetylase	Anti-inflammatory	Rothgiesser et al., 2010; Pais et al., 2013
Sirt3	Mitochondria	Deacetylase	Reduce oxidative stress, promote mitochondrial biogenesis, anti-inflammatory	Ansari et al., 2017; Jiang et al., 2017; Meng et al., 2019
Sirt4	Mitochondria	ADP-ribosyltransferase	–	–
Sirt5	Mitochondria	Desuccinylase, demalonylase, deacetylase	–	–
Sirt6	Nucleus	Deacetylase, ADP-ribosyltransferase	Neuroprotection	Jung et al., 2016; Kaluski et al., 2017; Mohamad Nasir et al., 2018
Sirt7	Nucleus (nucleolus)	Deacetylase	–	–

## Distribution of Sirtuins in Central Nervous System

Sirtuins are abundantly expressed in the brain (Jayasena et al., 2016), and Sirt2 has been identified as the most expressed of this enzyme family (Sidorova-Darmos et al., 2014). Several lines of evidence show that sirtuins are differentially expressed in different brain regions, and that their levels can change with age. Although Sirt1 expression has been reported to increase throughout the rat brain during aging, its deacetylase activity was found to be markedly declined in older animals. This reduced Sirt1 activity was suggested to be linked with the age-associated decline of its co-substrate, NAD^+^ in the brain (Braidy et al., 2015). However, Yan et al. (2019) recently demonstrated that Sirt1 expression in rat hippocampus is also decreased with age. Similarly, the expression of mitochondrial Sirts (Sirt3-5) decline significantly in the frontal lobe and hippocampus during aging (Braidy et al., 2015). Sirt1-3 are the predominant sirtuins identified in cultured neurons and glial cells, while Sirt1 and Sirt2 are detected in high levels in neurons and oligodendrocytes, respectively (Jayasena et al., 2016). A number of studies have revealed that the expression of Sirt1 and Sirt3 are reduced in the brain of the individuals with AD (Lutz et al., 2014; Lee et al., 2018; Yin et al., 2018). In addition, Sirt1 and Sirt3 have been detected in the blood, and their serum levels were shown to decline with age and were associated with frailty in humans (Kumar et al., 2014). Furthermore, a significant decrease in serum Sirt1 has been observed in AD patients (Kumar et al., 2013). Additionally, Sirt6 expression is found to decrease both in the brains of individuals with AD and in the mouse models of AD (Jung et al., 2016; Kaluski et al., 2017).

## Sirtuins and Alzheimer’s Disease

Sirtuins play important role in maintaining neuronal health during aging (Herskovits and Guarente, 2014) and accumulating evidence demonstrates that Sirts regulate multiple processes associated with the pathogenesis of AD, such as APP processing, tau aggregation, mitochondrial dysfunction, oxidative stress, and neuroinflammation (Lalla and Donmez, 2013; Jęśko et al., 2017; Lee et al., 2018; Mohamad Nasir et al., 2018; Rizzi and Roriz-Cruz, 2018). Sirt1 has been found to ameliorate AD pathology via a number of different mechanisms. Sirt1 decreases Aβ burden by promoting alternative processing of amyloid precursor protein, leading to the formation of non-amyloidogenic soluble APPα (sAPPα) (Qin et al., 2006; Lee et al., 2014; Zhang et al., 2020). The Sirt1 levels are reduced in the cerebral cortex of AD patients, and the decline of Sirt1 is correlated with the accumulation of Aβ and tau in the cerebral cortex (Julien et al., 2009). Moreover, caloric restriction, which is known to induce Sirt1 activity, has been shown to attenuate amyloid pathology in AD animals (Wang et al., 2005; Qin et al., 2006).

Post-translational modifications of tau, such as phosphorylation and acetylation play important roles in tau-induced neurodegeneration (Wang and Mandelkow, 2016; Guo et al., 2017). Acetylation prevents the degradation of phosphorylated tau and hence promotes tau accumulation and toxicity (Min et al., 2010; Cohen et al., 2011; Min et al., 2015; Tracy et al., 2016). Overexpression of Sirt1 has been demonstrated to reduce tau acetylation, resulting in attenuation of tau pathology in a mouse model of taupathy (Min et al., 2018). It has also been shown that Aβ increases the level of acetylated tau in transgenic mice via downregulation of Sirt3 expression (Yin et al., 2018). Tau acetylation decreases with Sirt3 overexpression, whereas tau acetylation increases after Sirt3 knockdown in HT22 mouse hippocampal neurons (Li et al., 2019). In addition, decreased Sirt3 levels lead to p53-mediated mitochondrial dysfunction and neuronal damage in AD (Lee et al., 2018).

Aβ has also been found to decrease the expression of Sirt6 (Jung et al., 2016), which is important in maintaining the genomic stability in the brain (Kugel and Mostoslavsky, 2014). Overexpression of Sirt6 has been demonstrated to offer protection against Aβ-induced DNA damage in hippocampal HT22 neuronal cells (Jung et al., 2016). Sirt6 also regulates tau phosphorylation by glycogen synthase kinase 3 (GSK3). Decreased brain Sirt6 is associated with increased tau phosphorylation via activation of GSK3 (Kaluski et al., 2017).

## Role of Sirtuins in Neuroinflammation

Growing evidence supports a significant role for sirtuins in the regulation of neuroinflammation. While the molecular mechanisms involved in microglial activation are currently under active investigation, emerging mechanisms of Sirts in the neuroinflammation process mainly focus on the microglial NF-κB signaling and inflammasome pathways (Figure 2).

### NF-κB Pathway

Sirt1 is the most studied mammalian sirtuin, which is known to suppress the inflammatory response through multiple mechanisms. Sirt1 inhibits the transcriptional activity of NF-κB via deacetylation of p65 (RelA) subunit at Ac-Lys310 (Yeung et al., 2004). Stimulation of microglia with Aβ_42_ peptide increases the acetylation of p65. Overexpression or pharmacological activation of Sirt1 elicits a neuroprotective effect against Aβ toxicity by inhibiting NF-κB signaling in microglia (Chen et al., 2005; Yang et al., 2012). In addition, upregulation of IL-1β transcription in microglia of old mice and in blood cells of aging humans and demented patients with tauopathy has been revealed to be associated with selective hypomethylation of IL-1β promoter (Cho et al., 2015). Sirt1 is also a regulator of DNA methyltransferases (DNMTs) that evoke epigenetic transcriptional repression by introducing methyl groups at CpG sites in DNA (Peng et al., 2011; Heo et al., 2017). Sirt1 in microglia has been shown to inhibit IL-1β expression by activating DNMT1 via deacetylation, thereby promoting the methylation of specific sites on IL-1β proximal promoter (Cho et al., 2015). The age-dependent decline of Sirt1 is associated with selective promotor hypomethylation, resulting in increased IL-1β transcription (Cho et al., 2015). Moreover, Sirt1 deficiency is reported to be associated with downregulation of DNMT1 expression in microglia of rats with anesthesia/surgery induced neuroinflammation, while the activation of Sirt1 with resveratrol prevents the repression of DNMT1 activity (Yan et al., 2019).

Sirt2 is the only mammalian sirtuin member primarily found in the cytoplasm, and is also the predominantly expressed sirtuin in the brain. Sirt2 can suppress microglial inflammatory and neurotoxic responses through deacetylation of Ac-Lys310 on p65 (Rothgiesser et al., 2010; Pais et al., 2013). Furthermore, Sirt2 has been identified as an inhibitor of the neuroinflammatory response associated with traumatic brain injury (TBI). Inhibition of Sirt2 enhances TBI-induced microglial activation and the release of proinflammatory cytokines via acetylation dependent upregulation of NF-κB transcriptional activity (Yuan F. et al., 2016). Overexpression of Sirt2 has been demonstrated to decrease neuroinflammation in a rat model of chronic constriction injury through deacetylation-induced inhibition of NF-κB signaling (Zhang and Chi, 2018). In contrast, several recent studies have suggested that Sirt2 can also promote inflammation. Treatment with the Sirt2 inhibitor AGK2 was found to block LPS-induced nuclear translocation of NF-κB and the expression of TNF-α and IL-6 in BV2 microglia and in a mouse model of neuroinflammation, indicating that Sirt2 is required for LPS-induced microglial activation and neuroinflammation (Wang et al., 2016). Thus, AGK2 treatment could significantly reduce the production of both NO and TNF-α in N9 microglia treated with LPS through the reduction of microglial activation (Harrison et al., 2018). Moreover, inhibition of Sirt2 has been found to improve α-synuclein toxicity in Parkinson’s disease models (Outeiro et al., 2007; Chen et al., 2015). Similarly, inhibition of Sirt2 resulted in cognitive improvements in two AD mouse models due to the suppression of Aβ production (Biella et al., 2016). Therefore, further investigations are required to clarify the effect of Sirt2 modulators in neuroinflammation associated with Alzheimer’s disease.

### Oxidative Stress and Inflammasome Pathway

Among the mitochondrial Sirts, Sirt3 has a number of effects on mitochondrial function, with the most significant being the suppression of oxidative stress (Ansari et al., 2017; Meng et al., 2019). Sirt3 expression has been found to be downregulated in response to LPS-induced inflammatory response in BV-2 microglial cells, besides LPS-mediated mitochondrial dysfunction, neuroinflammation, and microglial death can each be prevented by overexpression of Sirt3 (Zhou and Jiang, 2019). The inflammatory cytokines inhibit proliferation and promote apoptosis of neural stem cells (NSCs). Using a microglia-NSCs co-culture system it has been demonstrated that Aβ induced microglia activation leads to the accumulation of ROS, through the downregulation of Sirt3 and the antioxidant enzyme manganese superoxide dismutase (MnSOD) in NSCs. The overexpression of Sirt3 in NSC provided protection against microglial-derived, cytokine-induced neuronal death (Jiang et al., 2017). Recently, it was also reported that Sirt3 protects against anesthesia/surgery-induced cognitive decline by suppressing hippocampal neuroinflammation. In aged mice with postoperative cognitive dysfunction, microglial activation and elevated proinflammatory cytokine levels were found to be associated with decreased SOD and Sirt3 expression in the hippocampus (Liu et al., 2021). In addition, Sirt3 promoted microglial migration to the site of the lesion by upregulating the fractalkine receptor CX3CR1 in mice with ischemic stroke (Cao et al., 2019).

Inflammasomes are a key component of innate immunity, and the NLRP3 inflammasome is known to mediate neuroinflammation in Alzheimer’s disease. Inhibition of NLRP3 can hinder AD pathology and ameliorate cognitive impairment (Venegas and Heneka, 2019). Several studies have shown that the Sirt1 activator resveratrol alleviates different brain pathologies in rodents by inhibition of NLRP3 inflammasome activation, at least partly via Sirt1-mediated suppression of ROS production (Zhang X. et al., 2017; Zou et al., 2018). Sirt1 is also found to suppress CD40 mediated activation of NLRP3 inflammasome in vascular endothelial cells (Li et al., 2017). In addition, it has been shown that Sirt2 can inhibit NLRP3 inflammasome activation by two different mechanisms. The sirtuin activator resveratrol promotes Sirt2-dependent deacetylation of α-tubulin, resulting in suppression of acetylated α-tubulin mediated assembly of NLRP3 inflammasome in primary mouse macrophages (Misawa et al., 2015). In addition, He et al. (2020) recently reported that Sirt2 directly deacetylates conserved lysine residues located in the pyrin domain of NLRP3, thereby suppressing the inflammasome assembly in macrophages.

ROS and DAMPs, such as mitochondrial DNA released from damaged mitochondria, trigger the formation of the NLRP3 inflammasome (Zhou et al., 2011; Wilkins et al., 2017). There is substantial evidence to show that mitochondrial dysfunction and the resulting oxidative imbalance (i.e., oxidative stress) in the brain are associated with AD (Manoharan et al., 2016; Kausar et al., 2018; Llanos-González et al., 2020). Mitochondria, the power house of the cell, serves as a major source of ROS (e.g., superoxide radicals, hydroxyl radicals) in living systems. Accumulation of these highly reactive oxygen species primarily damage mitochondrial components, resulting in mitochondrial dysfunction (Bhat et al., 2015). Sirt3 is known to protect mitochondria against oxidative stress by deacetylating the transcription factor forkhead box O 3a (FoxO3a), which transactivates the antioxidant genes catalase and MnSOD, leading to detoxification of ROS (Rangarajan et al., 2015). Similarly, Sirt1 is also known to induce an antioxidant response via deacetylation of FoxO3a (Brunet et al., 2004). Moreover, Sirt3 directly activates MnSOD by deacetylation (Tao et al., 2014). A significant induction of Sirt3 expression has been observed in LPS-activated microglia in rat brain subjected to TBI. Knockdown of Sirt3 in microglia resulted in a decrease expression of MnSOD and associated increases in ROS levels. By contrast, overexpression of Sirt3 increased the expression of the antioxidant enzymes catalase and MnSOD (Rangarajan et al., 2015). In another study it was shown that the enzymes of the mitochondrial redox pathway, including MnSOD, were downregulated in the Sirt3^–/–^ mouse brain, and that the Sirt3 deficiency resulted in mitochondrial dysfunction, accumulation of ROS, and assembly of inflammasome in the brain leading to increased production of IL-1β (Tyagi et al., 2018). Taken together, both Sirt1 and Sirt3 inhibit NLRP3 inflammasome activation in microglia, most likely by suppressing the oxidative stress and mitochondrial damage. In contrast, Sirt2 inhibits NLRP3 inflammasome activation in macrophages through direct deacetylation of NLRP3 and α-tubulin. Nevertheless, the effects of Sirt2 on the activation of NLRP3 inflammasome in microglia have yet to be investigated.

The NLRP3 inflammasome is also activated by particulate matter such as monosodium urate, silica, alum, as well as by Aβ. These insoluble materials are likely to cause phagolysosome damage, leading to leakage of its contents into the cytosol (Kelley et al., 2019). Internalized Aβ elicits phagolysosomal damage and the liberated lysosomal protease, cathepsin B can activate the NLRP3 inflammasome in microglia (Halle et al., 2008; Wu et al., 2013; Campden and Zhang, 2019). Furthermore, mitochondrial ROS have also been demonstrated to cause lysosomal membrane damage and hence trigger the activation of the inflammasome (Heid et al., 2013). Therefore, strategies that can inhibit NLRP3 inflammasome activity in microglia might be effective in mitigating the chronic inflammation associated with aging and, significantly, could be beneficial in preventing AD.

## Sirtuin Activators for the Prevention of Alzheimer’s Disease

There are no effective therapies yet developed for the prevention or treatment of Alzheimer’s disease. Currently approved medications can result in some improvements in cognitive functions and behavioral symptoms, but do not slow the progression of AD pathology. It has previously been documented that modulation of sirtuin activity could be used as a treatment modality for many diseases, including neurodegenerative diseases, tumors, and metabolic syndrome. As discussed above, sirtuins play a central role in the brain homeostasis, but their expression/activity decline with age. Thus, restoration of sirtuin function in brain can potentially ameliorate the progression of AD. Therapeutic activation of sirtuin is emerging as an active area of research in the field of age-related disorders. Further, numerous natural and synthetic small-molecule sirtuin activators have shown to activate Sirt1 via binding to an allosteric site near its catalytic domain (Hubbard et al., 2013).

Resveratrol, a known activator of Sirt1, has been reported to reduce Aβ pathology in AD mouse models through a number of mechanisms (Karuppagounder et al., 2009; Vingtdeux et al., 2010). This activator exerts its anti-inflammatory effects mainly by inhibiting the NF-κB signaling cascade and inhibiting activation of the NLRP3 inflammasome (Yang and Lim, 2014; Wang et al., 2017; Li et al., 2018; Feng and Zhang, 2019). Activation of Sirt1 by resveratrol and overexpression of Sirt1 significantly reduces NF-κB signaling, which is stimulated by Aβ in microglia (Chen et al., 2005). Resveratrol treatment has been shown to modulate neuroinflammation, resulting in a slowing of cognitive and functional decline in individuals with mild to moderate AD (Moussa et al., 2017). In a diabetic rat model with concurrent AD, resveratrol lowers the cortical and hippocampal levels of IL-1β and IL-6 by increasing the expression of Sirt1 in the brain (Ma et al., 2020). Furthermore, resveratrol-induced Sirt1 activation attenuated the inflammatory responses in the cerebral cortex in rats with TBI by suppressing NLRP3 inflammasome-caspase 1 activation and ROS production. These beneficial effects of resveratrol were blocked by inhibiting Sirt1 activity with sirtinol (Zou et al., 2018). LPS decreases the expression of Sirt1 and increases the levels of TNF-α and IL-6 in activated BV-2 microglial cells, and these effects could be suppressed with resveratrol treatment (Ye et al., 2013). Moreover, in a recent study resveratrol was shown to increase the expression of both Sirt1 and Sirt3 in lymphocytes (Cosín-Tomàs et al., 2019). Resveratrol is a promising candidate for further testing in clinical trials as it has been found to be safe and well-tolerated up to 5 g/day in healthy individuals (Patel et al., 2011).

In addition to resveratrol, several classes of plant polyphenols, such as butein, isoliquiritigenin, piceatannol, and synthetic molecules like SRT1720 have been recognized as Sirt1 activators that could bring about neuroprotective effects (Dai et al., 2018). Butein and isoliquiritigenin attenuate H_2_O_2_-induced oxidative stress by upregulating the expression of Sirt1, FOXO3a, ADAM10, and the antioxidant enzymes catalase and MnSOD in human dopaminergic SH-SY5Y cells. ADAM10 is involved in processing of APP, which produces non-amyloidogenic sAPPα (Gay et al., 2020). Piceatannol, an analog of resveratrol, increases Sirt1 expression in THP-1 human monocytic cells (Kawakami et al., 2014). Sirt1 activation with SRT1720 ameliorates vascular endothelial dysfunction in aging mice, by decreasing NF-κB acetylation and subsequent reduction of oxidative stress and vascular inflammation (Gano et al., 2014). In addition, SRT1720 alleviates inflammation in an ovalbumin-induced mouse model of asthma by suppressing splenocyte proliferation and proinflammatory TNF-α and IL-6 production (Ichikawa et al., 2013).

Honokiol, a poly-phenolic compound with known neuroprotective properties, has now been identified as a Sirt3 activator (Woodbury et al., 2013). Honokiol enhances the antioxidant activity and mitochondrial function, and reduces Aβ generation through upregulating Sirt3 expression in Chinese hamster ovarian cells carrying the amyloid precursor protein and presenilin PS1 mutation (PS70 cells) (Ramesh et al., 2018). Honokiol attenuates surgery/anesthesia-induced cognitive impairment in mice through upregulating Sirt3 expression and thereby decreases oxidative stress and neuroinflammation in the hippocampus. 3-TYP, a Sirt3 inhibitor abolishes the neuroprotective effects of honokiol, indicating that honokiol exerts its effect via Sirt3 activation (Ye et al., 2019).

MDL-811, a recently discovered small-molecule activator of Sirt6, functions as an inhibitor of inflammation in LPS-stimulated mouse macrophages and microglia. The activated Sirt6 was found to deacetylate the enhancer of zeste homolog 2 (EZH2), a histone methyltransferase and ameliorate the neuroinflammation in ischemic brain injury via EZH2/forkhead box C1 signaling pathway in microglia (He et al., 2021).

In addition, NAD^+^ precursors can also be used to enhance Sirts activity. Since sirtuins require NAD^+^ as a co-substrate, their activity depends on the cellular NAD^+^ availability. It has been demonstrated that NAD^+^ levels in multiple organs, including the brain, decreases with age and this decrease can affect sirtuin functions (Imai and Guarente, 2016). Mounting evidence demonstrates that supplementation of NAD^+^ precursors, such as nicotinamide, nicotinamide mononucleotide, and nicotinamide riboside ameliorates numerous age-associated disorders (Imai and Guarente, 2016; Lautrup et al., 2019). It was also recently shown that NAD^+^ augmentation with nicotinamide riboside inhibits neuroinflammation in AD mice (Hou et al., 2018). Human clinical trials are warranted to evaluate the efficacy of NAD^+^ precursors on neuroinflammation and AD. Intake of nicotinamide, nicotinamide mononucleotide, and nicotinamide riboside chloride have been found to be safe up to 3 g/day, 500 mg/day, and 300 mg/day, respectively, in healthy people (EFSA Panel on Nutrition et al., 2019; Hwang and Song, 2020; Irie et al., 2020).

## Conclusion and Future Perspectives

Neuroinflammation has now being acknowledged as an important pathophysiological feature of neurodegenerative disorders like Alzheimer’s disease. Therefore, every factor that is associated with neuroinflammation could be a potential target for the treatment of AD. Although early microglial activation is shown to be neuroprotective, the persistent production of proinflammatory and cytotoxic molecules by chronically activated microglia leads to neurodegeneration. Therefore, suppression of microglial activity has been considered as a potential treatment option for AD.

Over the past decade an increasing number of studies have demonstrated that there is a link between sirtuins and neuroinflammation, both in the aging brain and in neurodegenerative disorders. Furthermore, there has been a significant progress in understanding the mechanisms by which sirtuins modulate inflammatory reactions in CNS. In this review, we have examined the latest evidence concerning the role of sirtuins in neuroinflammation, mainly focusing on NF-κB, and NLRP3 inflammasome pathways in microglia.

While all seven sirtuins are expressed in the mammalian brain, only a limited number of studies have been conducted to elucidate their individual roles in neuroprotection. The age-related decline of Sirt activity has recently been documented and the activation of sirtuins, particularly Sirt1, Sirt2, Sirt3, and Sirt6, has been shown to have beneficial effects on preventing AD. The course of AD pathology is significantly affected both by Sirt1 expression and by Sirt1 activity. The mitochondrial Sirt3 inhibits oxidative stress and mitochondrial damage, which in return suppresses the activation of NLRP3 inflammasome. Hence, strategies that activate sirtuin pathways in aging microglia may offer a new therapeutic avenue for the prevention or treatment of AD. Improved understanding of the underlying molecular mechanisms of pathway activation and their effects on neuroinflammation, can guide an approach to designing better pharmacological sirtuin activators against Alzheimer’s disease.

## Author Contributions

YW designed the manuscript and prepared the figures. KF and YW wrote, edited the manuscript, and approved the final version. Both authors contributed to the article and approved the submitted version.

## Conflict of Interest

The authors declare that the research was conducted in the absence of any commercial or financial relationships that could be construed as a potential conflict of interest.

## Publisher’s Note

All claims expressed in this article are solely those of the authors and do not necessarily represent those of their affiliated organizations, or those of the publisher, the editors and the reviewers. Any product that may be evaluated in this article, or claim that may be made by its manufacturer, is not guaranteed or endorsed by the publisher.
